# Diversity and Interrelations Among the Constitutive VOC Emission Blends of Four Broad-Leaved Tree Species at Seedling Stage

**DOI:** 10.3389/fpls.2021.708711

**Published:** 2021-09-24

**Authors:** Anne Charlott Fitzky, Arianna Peron, Lisa Kaser, Thomas Karl, Martin Graus, Danny Tholen, Mario Pesendorfer, Maha Mahmoud, Hans Sandén, Boris Rewald

**Affiliations:** ^1^Department of Forest and Soil Sciences, Institute of Forest Ecology, University of Natural Resources and Life Sciences Vienna, Vienna, Austria; ^2^Institute of Atmospheric and Cryospheric Sciences, University of Innsbruck, Innsbruck, Austria; ^3^Institute of Botany, University of Natural Resources and Life Sciences Vienna, Vienna, Austria

**Keywords:** *Betula pendula*, *Carpinus betulus*, emission blends, *Fagus sylvatica*, *Quercus robur*, VOC pathways, volatile organic compounds

## Abstract

Volatile organic compounds (VOCs) emitted by plants consist of a broad range of gasses which serve purposes such as protecting against herbivores, communicating with insects and neighboring plants, or increasing the tolerance to environmental stresses. Evidence is accumulating that the composition of VOC blends plays an important role in fulfilling these purposes. Constitutional emissions give insight into species-specific stress tolerance potentials and are an important first step in linking metabolism and function of co-occurring VOCs. Here, we investigate the blend composition and interrelations among co-emitted VOCs in unstressed seedlings of four broad-leaved tree species, *Quercus robur, Fagus sylvatica, Betula pendula*, and *Carpinus betulus*. VOCs of *Q. robur* and *F. sylvatica* mainly emitted isoprene and monoterpenes, respectively. *B. pendula* had relatively high sesquiterpene emission; however, it made up only 1.7% of its total emissions while the VOC spectrum was dominated by methanol (∼72%). *C. betulus* was emitting methanol and monoterpenes in similar amounts compared to other species, casting doubt on its frequent classification as a close-to-zero VOC emitter. Beside these major VOCs, a total of 22 VOCs could be identified, with emission rates and blend compositions varying drastically between species. A principal component analysis among species revealed co-release of multiple compounds. In particular, new links between pathways and catabolites were indicated, e.g., correlated emission rates of methanol, sesquiterpenes (mevalonate pathway), and green leaf volatiles (hexanal, hexenyl acetate, and hexenal; lipoxygenase pathway). Furthermore, acetone emissions correlated with eugenol from the Shikimate pathway, a relationship that has not been described before. Our results thus indicate that certain VOC emissions are highly interrelated, pointing toward the importance to improve our understanding of VOC blends rather than targeting dominant VOCs only.

## Introduction

Plants emit over 30,000 different biogenic volatile organic compounds (VOCs) into the atmosphere (Trowbridge and Stoy, 2013). Chemically, VOCs are molecules of low molar mass, consisting of isoprenoids (isoprene, monoterpenes, and sesquiterpenes), oxygenated chemical species (OVOCs, e.g., methanol, ethanol, acetone, and acetaldehyde) and green leaf volatiles (GLVs; e.g., hexenals) (Oikawa and Lerdau, 2013). Among biogenic VOCs, isoprene accounts for ∼44% of all emissions, and monoterpenes and methanol each constitute ∼11% of all VOC emissions (Guenther et al., 1995; Guenther et al., 2006). Sesquiterpenes and a great number of other compounds are often emitted in lesser quantities (Duhl et al., 2008; Loreto and Schnitzler, 2010; Niinemets and Monson, 2013). Among vegetation components, woody species are generally considered a major source of VOCs (Rosenkranz and Schnitzler, 2013). However, type and rate of VOC emissions are highly species-specific. Species with high emission rates are thus frequently divided into isoprene, monoterpene, and sesquiterpene emitters, and distinguished from non- or “close-to-zero”-emitting species (see Niinemets and Monson, 2013 and references within; Dani et al., 2014). In addition, other VOCs emitted in larger quantities, such as methanol, are used for classification (Brunner et al., 2007). While common classification schemes focus on the emission rate of dominant VOCs (Karl M. et al., 2009; Dani et al., 2014) or single VOCs of (assumed) functional significance (Kanagendran et al., 2018; Werle et al., 2019), trees, as other plant species, emit several VOCs simultaneously—creating a diverse blend of compounds (Niinemets and Monson, 2013).

Primary functions of individual airborne VOCs have been related to the defense of plants against herbivores and pathogens, the attraction of pollinators, seed dispersers and other beneficial animals and microorganisms, and signaling in plant–plant interaction (Holopainen and Gershenzon, 2010; Oikawa and Lerdau, 2013). In addition, specific VOCs were reported to increase the tolerance for abiotic stresses, e.g., by detoxification of deposited photooxidation products, or outcompeting neighboring species *via* an induced growth-repression (Trowbridge and Stoy, 2013; Canaval et al., 2020). In addition to single VOC effects, evidence on the functional significance of VOC blends is increasing, suggesting that multiple VOCs emitted simultaneously can constitute modified, synergistic responses (Niinemets and Monson, 2013). For example, the wound-induced GLVs emitted by *Tanacetum cinerariifolium* activated the biosynthesis of insecticidal metabolites in neighboring plants effectively only as a mixture of several compounds (Ueda et al., 2012). Similar, only the blend of different terpenes and GLVs was considered effective as an herbivore warning system in *Betula nana* (Li et al., 2019). The attraction of predators in response to an herbivorous attack on *Festuca* spp. was not determined by the total amount of VOCs emitted, but only by a specific combination of several VOCs (and their ratios)—a larger number of predators were attracted by an increased concentration of this VOC blend (Kergunteuil et al., 2020). Earlier it was shown that different monoterpene blends alter the abiotic stress tolerance of both emitting and neighboring non-emitting species differently (Copolovici et al., 2005). While the diversity and quantity of VOC blends are increasingly studied and reported to vary widely across species and environmental conditions, information about the correlated emission of VOCs remains limited (Laule et al., 2003; Niinemets and Monson, 2013).

Four main interrelated metabolic pathways are thought be involved in the synthesis of major VOCs: methylerythritol phosphate (MEP), mevalonic acid (MVA), lipoxygenase (LOX) and shikimate (Laule et al., 2003; Dudareva et al., 2013). Other VOCs originate from catabolic processes, or reactions within the cell membranes (Peñuelas and Staudt, 2010; Oikawa and Lerdau, 2013; Dorokhov et al., 2018). Common derivatives and transcriptional factors linking those pathways and catabolic processes have yet to be studied in relation to their overlapping/combined functions (Li and Sharkey, 2013; Monson, 2013). An increased understanding of the interactions between jointly emitted volatile substances and its functional significance, however, is restrained because full VOC emission spectra are not yet available for a broad set of species. This is partially due to inherent technical difficulties related to detecting VOCs at low concentrations. However, recent advances of online proton-transfer-reaction time-of-flight mass spectrometry (PTR-TOF-MS) systems, in combination with dynamic/open chamber systems, now allow for measurements of VOC blends at high mass and time resolution (Graus et al., 2010). Absorbance and reactions between VOCs can now be minimized compared to closed chamber systems or absorbent tubes (Tholl et al., 2006; Kolari et al., 2012). These novel approaches can thus provide more insights into the pathways involved in VOC synthesis, as they allow of the simultaneous measurements of low-concentration VOCs.

As most previous studies have predominantly targeted single VOCs, or VOC spectra of single individuals or mixed-species plantations, identification of species-specific “VOC fingerprints” and their inter-specific comparability is hampered. However, detailed characterization of constitutive VOC blends under non-stress conditions is a prerequisite for the assessment of functional changes in volatile spectra under altered environmental conditions. The general aim of this study is thus to determine the constitutive VOC emission spectra of four common European tree species, allowing (1) to compare species-specific VOC blends (i.e., “fingerprints”), and (2) to identify co-emission pattern of different VOCs which may hint at the underlying metabolic pathways. The selected tree species represent four major types of emitters, commonly classified as isoprene- (*Quercus robur* L.), monoterpene- (*Fagus sylvatica* L.), sesquiterpenes- (*Betula pendula* Roth.), and close-to-zero-emitters (*Carpinus betulus* L.).

## Materials and Methods

### Species and Growth Conditions

Two-year-old seedlings of *Q. robur, F. sylvatica, B. pendula*, and *C. betulu*s were planted in 7 L pots filled with a 1:2 mixture of “Viennese soil substrate” (used by arborists for planting trees in Vienna; see Supplementary Materials and Methods for details) and quartz sand in March 2019. The species are major vegetation components in temperate forests across Europe (Ellenberg and Leuschner, 2010), and also frequently used for urban greenery (Pauleit et al., 2002; Sukopp and Wurzel, 2003). After planting, pots were fertilized using 50 g m^–2^ of a universal NPK fertilizer (8% N, 8% P_2_O_5_, 6% K_2_O, 0.02% Fe, 0.01% B and Mn, 0.002% Cu and Zn, and 0.001% Mo; NovaTec, Compo, Münster, Germany). Eight replicates per species were subsequently grown for 14–16 weeks in a greenhouse in Tulln (Austria) under well watered (∼100% field capacity, 13.4 vol.% water content; Fieldscout TDR100, 20 cm probe depth, Spectrum Technologies, United Kingdom) and near ambient light and temperature conditions. Two weeks before VOC sampling (June 2019), the seedlings had fully expanded and toughened leaves and were relocated to a ventilated greenhouse in Vienna and kept at near ambient temperature conditions. The shoots were carefully rinsed with tap water 1 week before measurements, removing dust and fingerprints, and from thereon were only handled with cotton gloves. The trees were moved to a climate chamber (Fitotron, Weiss Gallenkamp, United Kingdom; 25°C air temperature, 40% RH) for acclimation at least 24 h before enclosing them into VOC-chambers for measurements.

### VOC Measurements

**FIGURE 1:**
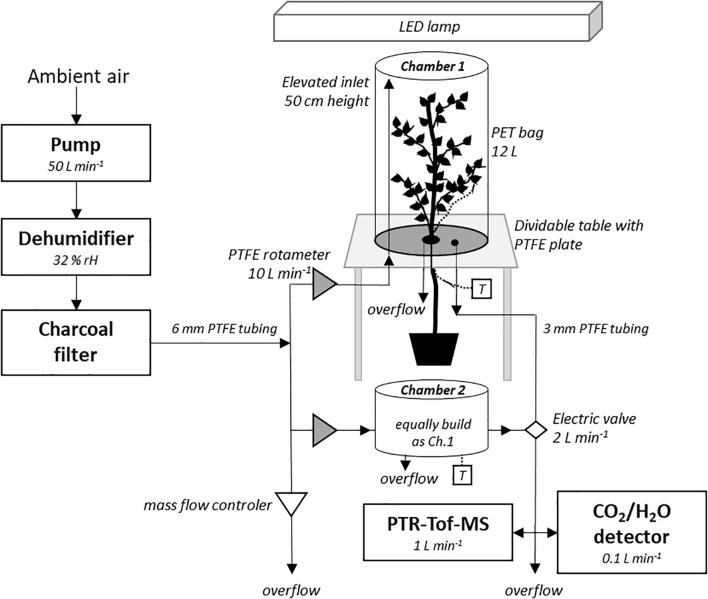
Setup of the experiment of the VOC chambers. Ambient air was flushed through the dehumidifier (32% RH) and the charcoal filter for providing close-to VOC-free air. The incoming air was entering each chamber through an elevated inlet. A mass flow controller was regulating the incoming air pressure. The PTFE-covered table was dividable, allowing for tree insertion, and sealed with a PTFE-coated silicon stopper. A tree was inserted into chamber 1, whereas chamber 2 remained empty for parallel VOC background measurements. A thermocouple (T) was installed at both chambers. A PET-bag was placed over the bottom plate and sealed. Two outlets in the bottom of the PTFE plates of each chamber were used as overflow and for gas analysis by the PTR-TOF-MS and CO_2_/H_2_O detector, respectively. See “Materials and Methods” for details, and Supplementary Figure 1 for images of the chamber.

The VOC emission blends were measured in June and July 2019 in an acclimatized growth room at 25°C in the morning and afternoon, 4 days a week. The gas sampling was performed using two custom-made VOC-chambers (TC-400, Vienna Scientific Instruments, Alland, Austria), consisting of a polyethylenterephthalat (PET)-bag (∼12 L, 55 × 60 cm, Malvern, United Kingdom) placed over a polytetrafluorethylen (PTFE, “Teflon”) plate with elevated PTFE-coated inlets (Figure 1 and Supplementary Figure 1). To insert the tree into chamber 1 (tree-chamber), the table with the PTFE plate was split open to place the seedling’s stem in a centered hole in the plate. Before closing the table and sealing the stem with a PTFE-coated silicon stopper, a thermocouple (type K, PTFE wire IEC; RS Pro thermocouple, RS components, Gmünd, Germany) was placed underneath a mid-canopy leaf per tree to monitor leaf temperature (°C). The PET-bag was placed over the inserted tree and sealed around the PTFE plate. The chamber 2 (control-chamber) stayed empty for parallel background measurements. A thermocouple was placed ∼10 cm above the PTFE plate to determine ambient air temperature. A membrane compressor (Hiblow HP 200, Takatsuki, Japan) was used for drawing ambient air from outside *via* suction, which was dried by a de-humidifier to ∼32% RH (VPD 2.386 kPa) before being passed through a charcoal filter (maximum flow 6000 L min^–1^; PrimaKlima, Radnice, Czechia) to reduce background-VOCs (Sidheswaran et al., 2012). The air was subsequently channeled using PTFE tubes and divided into one channel per chamber. Air entered each chamber at 50 cm above the PTFE plate (Figure 1); the air flow was adjusted by a PTFE valve (rotameter series 4L, emtechnik, Maxdorf, Germany) to 10 L min^–1^. Customized LED lights (Eckel electronics, Trofaiach, Austria) provided 47% red, 21% green, 20% blue, 11% far-red light with 1450 μmol m^–2^ s^–1^ at mid chamber height. Light intensity was measured beforehand in an empty chamber at different heights and calculated for each tree individual at mid canopy height. Each chamber had two outlets in the bottom plate, which were either used for (1) online-VOC measurements with a PTR-TOF-MS (PTR-TOF 6000 X2, IONICON Analytik GmbH, Innsbruck, Austria; Sulzer et al., 2014) and CO_2_/H_2_O gas measurements (IRGA CIRAS-3 DC/SC, PP-System, Amesbury, MA, United States) or (2) as an overflow (preventing over-pressure). Outlets (1) of the tree- and control-chambers were sequentially sampled by PTR-TOF-MS and IRGA through a set of electric valves (Figure 1).

Each tree was acclimated to the chamber environment for 2 h for stabilizing VOC emission rates. After 2 h, VOCs, CO_2_ and H_2_O were sampled twice for 5 min with a 15 min gap in between; means were calculated by combining both 5 min sampling periods. Detailed VOC emission calculations are listed in Supplementary Materials and Methods. The PTR-TOF-MS was operated in H_3_O^+^ mode at 350 V drift voltage, ion funnel settings of 1 MHz and 35 V amplitude as well as 35 VDC, drift pressure of 2.5 mbar and drift tube temperature of 100°C. It was calibrated daily using a VOC standard mixture (Apel Riemer Environmental Inc., Broomfield, CO, United States) containing 15 compounds following Peron et al. (2021). VOCs emitted by the tree-chamber and tubing were measured in the yet empty chamber (before enclosing a tree) and were subsequently subtracted from the VOC emission rates measured (see Supplementary Materials and Methods for details). Background VOCs of the incoming air (after passing the charcoal filter) were accounted for by the parallel, empty control-chamber (Figure 1).

We used the software Data Analyzer v.4 (Müller et al., 2013) to analyze PTR-TOF-MS results. Mass to charge ratios (*m/z)* were assigned to compounds as described in Peron et al. (2021); all the analyzed compounds are reported at the respective nominal *m/z*. Isoprene was detected at *m/z* 69, oxygenated products of isoprene methyl vinyl ketone (MVK) and methacrolein (MAC) at *m/z* 71, methyl ethyl ketone (MEK) at *m/z* 73, and 2-methyl-3-buten-2-ol (MBO) at *m/z* 87. The sum of monoterpenes (MT) was detected at *m/z* 137, and the sum of sesquiterpenes (SQT) at *m/z* 205. *M/z* 93 is indicative for toluene, but some monoterpenes show a minor fragment at that mass-to-charge ratio (Tani et al., 2003). Here, *m/z* 93 was associated to toluene emissions but the ambiguity is indicated throughout the text. GLVs were assigned to hexanal at *m/z* 101, hexenal at *m/z* 99, hexene at *m/z* 85, hexenyl acetate at *m/z* 143, and hexyl acetate at *m/z* 145. Shikimate BVOCs were tentatively assigned for benzene at *m/z* 79, benzaldehyde at *m/z* 107, methyl salicylate (MeSA) at *m/z* 153, and eugenol at *m/z* 165. Among oxygenated VOCs (OVOCs), methanol was assigned to *m/z* 33, ethanol to *m/z* 47, acetaldehyde to *m/z* 45, acetic acid to *m/z* 61, and acetone to *m/z* 59. Measurement precision, based on daily calibration, varied in the order of 8–20% depending on the compound (data not shown). Compounds with a concentration below <0.05 ppbv were considered not quantifiable (∼limit of quantification, LOQ). However, since the PTR-TOF-MS response is theoretically linear over the detected range, we extrapolated beyond the concentrations of the standard and report derived emission rates for these compounds. All emission rates were expressed per unit leaf area in nmol m^–2^ s^–1^. Light intensity and temperature recordings were used for calculating standardized VOC emissions for isoprene and acetaldehyde (Guenther et al., 1993), MT, and SQT emissions (Geron et al., 1994; see Supplementary Materials and Methods for details). All detected compounds were summed as total constitutive VOC emissions per species and expressed as absolute and relative values. VOC emission rates of “low concentration” VOCs, i.e., excluding the main emitted VOCs (isoprene for *Q. robur*, monoterpenes for *F. sylvatica*, and methanol for *B. pendula* and *C. betulus*) were calculated for comparison.

CO_2_ and H_2_O concentrations and leaf temperature in the chambers were used to calculate assimilation rate and stomatal conductance — characterizing plant physiological activity in parallel to the VOC measurements. In brief, the difference of gas concentrations in the tree-chamber and empty control-chamber during the VOC measurements and gas flow rates were used, following the manufacturer’s guidelines (PP-Systems, 2018); see Supplementary Materials and Methods for details.

### Statistical Analyses

The data collected with the PTR-TOF-MS was processed and averaged over the sampling time periods with MATLAB (MATLAB and Statistics Toolbox Release 2017; The MathWorks, Inc., Natick, MA, United States). Statistical analysis of assimilation rate, stomatal conductance, and VOC emissions were performed using R 4.0.5 (R Core Team, 2020). The mean and SD were calculated for all VOC emissions per species. To investigate species differences of normally distributed data (i.e., log-transformed SQT and total VOC emissions, and stomatal conductance), one-way analysis of variance (ANOVA) and Tukey’s HSD test were performed. Relative emission rates were calculated and visualized using the R package “ggplot2” (Wickham, 2016). To determine whether VOC emission patterns co-varied along major axes across and within species, e.g., due to shared pathways, we conducted a principal component analysis (PCA) using R packages “factoextra” (Kassambara and Mundt, 2017) and “ggbiplot” (Wickham, 2016). Pearson correlations were conducted to determine correlations between single VOC emissions using R package “corrplot” (Wei et al., 2017). Across the manuscript, similar colors were used for VOCs related to specific pathways and/or identified as OVOCs.

## Results and Discussion

### Composition of VOC Blends

Our study found different VOC blends for *Q. robur, F. sylvatica, B. pendula*, and *C. betulus* at seedling stage (Table 1 and Supplementary Tables 2, 3). A total of 22 *m/z* were detected above background concentrations in a dynamic VOC-chamber system with an online PTR-TOF-MS (Figure 1). Throughout the sampling period, neither diurnal (morning, afternoon) nor weekly (June to July) changes of VOC emissions were observed (data not shown). While stomatal conductance partly regulates the constitutive emissions depending on the VOC’s volatility (Grote et al., 2013), stomatal conductance did not differ significantly between species. Assimilation rates suggest that the trees were not stressed (Supplementary Table 1).

**TABLE 1:** Constitutive VOC emission rates (nmol m^–2^ s^–1^) of *Q. robur, F. sylvatica, B. pendula*, and *C. betulus* seedlings (Mean ± SD).

***m/z* ratio**	**Chemical formula**	**Assigned compound(s)**	Constitutive VOC emission rates (nmol m^–2^ s^–1^)*			
			***Quercus robur***	***Fagus sylvatica***	***Betula pendula***	***Carpinus betulus***
**Oxygenated VOCs**						
33.034	(CH_4_O)H^+^	Methanol	0.039 ± 0.115	0.061 ± 0.111	0.913 ± 0.571	0.313 ± 0.372
47.049	(C_2_H_6_O)H^+^	Ethanol	0.002 ± 0.002	0.004 ± 0.008^§^	0.004 ± 0.003	0.011 ± 0.013
45.034	(C_2_H_4_O)H^+^	Acetaldehyde^*Std*^	0.018 ± 0.008	0.023 ± 0.010	0.025 ± 0.010	0.020 ± 0.007
61.028	(C_2_H_4_O_2_)H^+^	Acetic acid	0.010 ± 0.003	0.015 ± 0.003	0.043 ± 0.020	0.014 ± 0.003
59.049	(C_3_H_6_O)H^+^	Acetone	0.037 ± 0.025	0.012 ± 0.016^§^	0.129 ± 0.020	0.013 ± 0.007
**LOX-pathway**						
101.096	(C_6_H_12_O)H^+^	Hexanal	0.0002 ± 0.0001^§^	0.0001 ± 0.0001^§^	0.0006 ± 0.0002^§^	0.0003 ± 0.0001^§^
99.080	(C_6_H_10_O)H^+^	Hexenals	0.0005 ± 0.0001	0.0005 ± 0.0002^§^	0.001 ± 0.001^§^	0.0010 ± 0.0003^§^
85.101	(C_6_H_12_)H^+^	Hexene	0.0004 ± 0.0003^§^	0.0002 ± 0.0002^§^	0.0002 ± 0.0002^§^	0.0001 ± 0.0002^§^
143.107	(C_8_H_14_O_2_)H^+^	Hexenyl acetate	0.0010 ± 0.0002	0.0010 ± 0.0002^§^	0.0010 ± 0.0003^§^	0.0010 ± 0.0002^§^
145.122	(C_8_H_16_O_2_)H^+^	Hexyl acetate	0.0001 ± 0.0001^§^	0.0001 ± 0.0002^§^	0.0002 ± 0.0001^§^	0.0002 ± 0.0001^§^
57.069	(C_4_H_8_)H^+^	Butyl	0.005 ± 0.004	0.001 ± 0.002^§^	0.002 ± 0.001^§^	0.001 ± 0.001^§^
**MEP-pathway**						
69.069	(C_5_H_8_)H^+^	Isoprene^Std^	20.831 ± 4.837	0.008 ± 0.004	0.019 ± 0.007	0.007 ± 0.003
71.049	(C_4_H_6_O)H^+^	MVK/MAC	0.004 ± 0.001	0.001 ± 0.001^§^	0.003 ± 0.001	0.0010 ± 0.0003^§^
73.065	(C_4_H_8_O)H^+^	MEK	0.006 ± 0.007	0.004 ± 0.007^§^	0.011 ± 0.005	0.003 ± 0.003^§^
87.080	(C_5_H_10_O)H^+^	MBO	0.017 ± 0.004	0.005 ± 0.002	0.011 ± 0.003	0.006 ± 0.001
137.132	(C_10_H_16_)H^+^	Sum of MTs^Std^	0.029 ± 0.027	0.390 ± 0.359	0.080 ± 0.030	0.072 ± 0.110
93.069	(C_7_H_8_)H^+^	F MT/toluene	0.001 ± 0.001	0.006 ± 0.005	0.002 ± 0.001^§^	0.001 ± 0.001^§^
**MVA-pathway**						
205.195	(C_15_H_24_)H^+^	Sum of SQTs^Std^	0.007 ± 0.011	0.013 ± 0.012	0.022 ± 0.021	0.006 ± 0.003^§^
**Shikimate-pathway**						
79.054	(C_6_H_6_)H^+^	Benzene	0.0010 ± 0.0003	0.001 ± 0.001^§^	0.001 ± 0.001^§^	0.001 ± 0.001^§^
107.049	(C_7_H_6_O)H^+^	Benzaldehyde	0.0010 ± 0.0001	0.0005 ± 0.0002^§^	0.0006 ± 0.0002^§^	0.0010 ± 0.0002^§^
153.055	(C_8_H_8_O_3_)H^+^	Methyl salicylate	0.002 ± 0.002	0.003 ± 0.002^§^	0.003 ± 0.002^§^	0.047 ± 0.053
165.092	(C_10_H_12_O_2_)H^+^	Eugenol	0.0001 ± 0.0001^§^	0.0001 ± 0.0001^§^	0.0002 ± 0.0001^§^	0.00010 ± 0.00003^§^
**TOTAL VOC**			21.383 ± 5.054	0.552 ± 0.546	1.295 ± 0.700	0.525 ± 0.584

*Mass-to-charge ratios (*m/z*) are assigned to VOCs: their chemical formula and the compound name. The measured VOCs are sorted in oxygenated VOCs, and VOCs synthesized by the LOX-, MEP-, MVA-, and Shikimate pathways and the respective catabolites, respectively. See Supplementary Tables 2, 3 for ANOVA and Tukey HSD test, respectively, on SQT and total VOC emissions.*

*F, fragment; MBO, 2-methyl-3-buten-2-ol; MVK, methyl vinyl ketone; MAC, methacrolein; MEK, methyl ethyl ketone; MT, monoterpenes; SQT, sesquiterpenes.*

*^Std^Standardized emission rates of isoprene, the sum of monoterpenes and sesquiterpenes, and acetaldehyde are marked with “Std.”*

*Concentrations under the limit of quantification (LOQ < 0.05 ppbv) are marked with an “§.”

**FIGURE 2:**
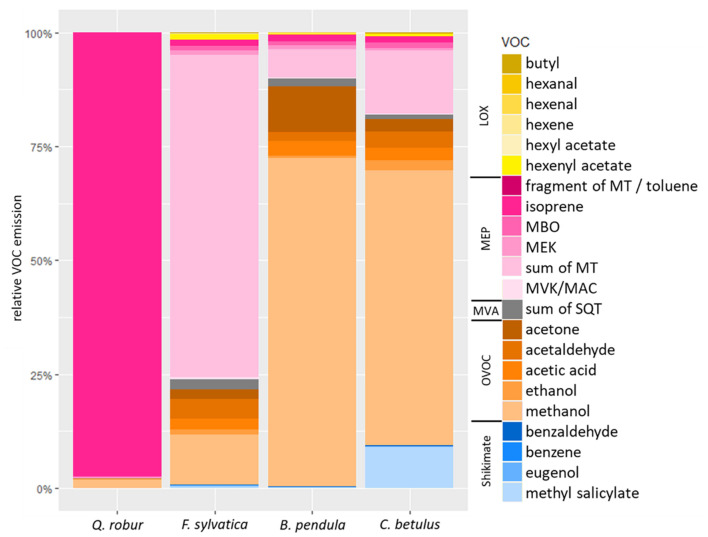
Relative constitutive VOC emission rates (%) of *Q. robur, F. sylvatica, B. pendula*, and *C. betulus* seedlings. VOCs are color-coded as being related to LOX- (yellow), MEP- (magenta), MVA- (gray), and Shikimate (blue) pathways or being oxygenated VOCs (OVOCs; orange). MBO, 2-methyl-3-buten-2-ol; MVK, methyl vinyl ketone; MAC, methacrolein; MEK, methyl ethyl ketone; MT, monoterpenes; SQT, sesquiterpenes. See Table 1 for VOC emission rates.

The main constitutive VOC emitted by *Q. robur* was isoprene (20.83 μmol m^–2^ s^–1^, 97.5% of total VOC emission), while monoterpenes (0.39 μmol m^–2^ s^–1^, 70.9% of total VOC emission; Table 1 and Figure 2) dominated the emission spectra of *F. sylvatica*, confirming previous reports (Karl M. et al., 2009; van Meeningen et al., 2016; Peron et al., 2021). *B. pendula* emitted significantly greater amounts of sesquiterpenes compared to *Q. robur* (*p* < 0.05; Table 1 and Supplementary Table 3). The recorded sesquiterpene emissions were 50% higher than previously reported (König et al., 1995; Duhl et al., 2008), but 90% lower than reported by Karl M. et al. (2009). However, sesquiterpene emissions contributed only 1.7% to *B. pendula*’s emission spectra, which was dominated by methanol (0.93 μmol m^–2^ s^–1^, 72.2%). The proposed “close-to-zero emitter” *C. betulus* (Karl M. et al., 2009) emitted VOCs in similar amounts as *F. sylvatica* and *B. pendula* (Table 1); the main emitted VOC was also methanol (0.31 μmol m^–2^ s^–1^, 60.3%; Table 1 and Figure 2). While elevated methanol emissions were often detected in above-canopy measurements of ecosystems containing *C. betulus* (Acton et al., 2016; Schallhart et al., 2016), specific methanol emission rates of *C. betulus* were, to the best of our knowledge, not previously reported, or even explicitly not detected among OVOCs (König et al., 1995). Our data thus constitutes that *B. pendula* and *C. betulus* seedlings dominantly emit OVOCs, specifically methanol. Monoterpene emissions of *C. betulus* constitute 13.8% of released VOCs (Figure 2), confirming earlier reports (Karl M. et al., 2009; Zemankova and Brechler, 2010).

Out of the 22 detected VOCs, 18 were emitted at rates above LOQ by *Q. robur* (99.6% of total emissions), 7 by *F. sylvatica* (94.4%), 11 by *B. pendula* (99.1%), and 11 by *C. betulus* (96.9%; Table 1 and Figure 2). Excluding the mainly emitted VOCs discussed above, other VOCs accounted for 2.5% of total VOC emissions in *Q. robur* (0.55 μmol m^–2^ s^–1^) only, but amounted to ∼29% of total emissions in *B. pendula* and *F. sylvatica* (0.36 and 0.16 μmol m^–2^ s^–1^, respectively). In *C. betulus*, emissions of these “less frequent” VOCs even accounted for ∼40% (0.21 μmol m^–2^ s^–1^) of total emissions (Table 1 and Figure 2). Note that the proportion of non-quantifiable VOCs (<LOQ) was slightly greater in *F. sylvatica* and *C. betulus* than in the other two species, ranging between 3 and 5% of total emissions. For example, sesquiterpene concentrations were not quantifiable in *C. betulus*, contradicting high emission rates reported earlier (König et al., 1995). In turn, emissions of some GLVs (i.e., hexenal, hexenyl acetate, and butyl) were only quantifiable in *Q. robur* (Table 1). In contrast, two elevated emission rates in specific species are noteworthy: acetone emissions were particularly high in *B. pendula* (10.1% of total emission) while being rather low for other species (<2.6%). Similarly, MeSA constituted 9.1% of *C. betulus* total emissions, i.e., MeSA emission rates being ∼15 times greater than in the other tree species (Figure 2). Among the further VOCs emitted by all species at low rates, for example, ethanol, acetaldehyde, and acetic acid constituted <4.1% of total emissions, while MBO accounted for <1.2% of total emissions (Table 1 and Figure 2).

### VOC Mapping to Metabolic Pathways, Catabolic Processes, and Known Functions

Volatile organic compounds are produced by all plant parts. However, as flowers were absent in the measured trees, synthesis of VOCs likely took place in mesophyll cells with release through stomata or cuticle (Loreto and Schnitzler, 2010). Some VOCs, however, may be released by the shoot after translocation from the root system (Delory et al., 2016). The detected VOCs are associated with either MEP-, MVA-, LOX-, and Shikimate pathways or are OVOCs derived from catabolic processes as discussed below. For a schematic overview on the interrelation of biochemical pathways related to VOC emissions see Maffei (2010).

Seven VOCs are related to the **MEP-pathway** (Table 1). Isoprene and monoterpenes were emitted by all species, as discussed above, and serve, e.g., as antioxidants under ozone and heat stress (König et al., 1995; Oikawa and Lerdau, 2013). MBO was emitted by all tree species at low rates (Table 1 and Figure 2). As isoprene, MBO is formed from the precursor DMAPP (dimethylallyl diphosphate) and has antioxidant functions (Lichtenthaler, 2007; Jardine et al., 2013). In contrast, MVK, MAC, and MEK are photo-oxidative products of isoprene and occur in high quantities at high ozone levels (Karl T. et al., 2009; Fares et al., 2015). Congruously, they were emitted by all studied tree species at rather low rates. Last, toluene (*m/z* 93), was emitted in quantifiable amounts by *F. sylvatica* and *Q. robur* (Table 1). The function and origin of toluene is still uncertain, particularly if it is synthesized by the MEP, MVA- or Shikimate-pathway (Heiden et al., 1999; Misztal et al., 2015). However, as *m/z* 93 may potentially comprise also a monoterpene fragment (in particular in the distinct MT emitter *F. sylvatica*), toluene has been assigned here as MEP-related.

Sesquiterpenes are synthesized by the **MVA-pathway** and were detected in all species. Sesquiterpenes constitute many different compounds, however, the SQTs blend emitted from herbivore- or phytopathogen-infested plants is often more diverse than of healthy plants (Mofikoya et al., 2019 and references within). As sesquiterpene emissions are often herbivore-induced, it is not surprising that SQTs were emitted at relatively low rates. The herbivore-free conditions of our experiment may also explain why SQT concentrations were not quantifiable in *C. betulus*, in contrast to earlier findings reporting high emission rates *in situ* (König et al., 1995).

Six different GLVs related to the **LOX-pathway** were identified and detected in all tree species. Since GLVs are mainly emitted in response to herbivory or other mechanical damage of leaf tissue, their constitutive emission rates could be expected to be low (Holopainen and Blande, 2013; Scala et al., 2013). However, among the plants initially established (i.e., at Tulln greenhouse) we observed few *Q. robur* individuals with a slight infection of powdered mildew (*Erysiphe alphitoides*), known to increase emissions of LOX-originated VOCs (Copolovici et al., 2014). While all individuals with visible infection signs were rigorous excluded from further use, we cannot completely preclude that some *Q. robur* plants were at an early stage of infection. This may have already induced elevated emissions of some GLVs (i.e., hexenal, hexenyl acetate, and butyl) in *Q. robur* (Table 1).

Four VOCs synthesized by the **Shikimate-pathway** were observed, mostly in low to very low concentrations (Table 1). MeSA emissions increase the stress tolerance against wounding, pathogen infestation and salt stress (Weaver and Herrmann, 1997; Zhang et al., 2011). Alternatively, benzenoids such as benzaldehyde, eugenol, and MeSA were previously related to insect attraction (Guerrieri, 2016; Junker, 2016; Binyameen et al., 2018). As neither such stressors nor flowers were present on the tested individuals, further studies are required to determine potential triggers, and the functional significance, of elevated MeSA emissions in *C. betulus*.

As methanol is the third most abundant VOC emitted by vegetation (Messina et al., 2016), it does not surprise that methanol is a key VOC within the emission blends of all four species—dominating in particular among the six **OVOC** compounds (Table 1). Since methanol is released during demethylesterification of cell wall pectins (Fall and Benson, 1996; Dorokhov et al., 2018), large methanol emissions may indicate rapid cell wall expansion and restructuring during growth (Niinemets et al., 2011). The greater juvenile growth rate of seedlings, particular of the fast-growing pioneer species *B. pendula*, may underlie the detected high methanol emissions compared to mature trees. Similar, we can only speculate that the greater acetone emissions of *B. pendula* were related to a high growth rate associated with changes among cellular membranes (Schade and Goldstein, 2006). Alternatively, cutting and/or fatty acid degradation have previously been shown to underly increased acetone emissions (Macdonald and Fall, 1993; De Gouw et al., 2000; Warneke et al., 2002). Ethanol, which is produced during low oxygen events by ethanol fermentation, can be upregulated during oxidative stress and can be transported from the roots into the leaves, where it is oxidized to acetaldehyde and eventually to acetic acid (Oikawa and Lerdau, 2013). During stress events, ethanol offers a fast C-source to be incorporated into steroids and fatty acids and is therefore catabolized to acetic acid (Kesselmeier and Staudt, 1999). Because the soil was watered to field capacity, hypoxic conditions resulting from water logging were unlikely to occur and the source of ethanol remains unknown. Acetaldehyde emissions can occur as a product of ethanol oxidation, and as a byproduct due to a release of excess pyruvate during dark/shaded conditions, whereas further origins are unknown (Karl et al., 2002).

Across the contrasting tree species, emitted VOCs were dominantly synthesized by the MEP-pathway or were OVOCs. *C. betulus*, however, additionally emitted larger quantities of MeSA, related to the Shikimate-pathway (Figure 2).

### Co-emission Pattern Among VOCs

**FIGURE 3:**
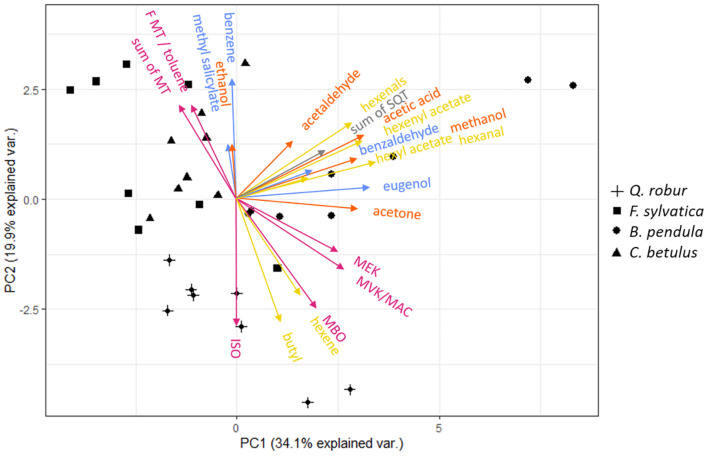
Principal component analysis (PCA) of 22 *m/z* assigned to constitutive VOC emissions of *Q. robur* (cross), *F. sylvatica* (square), *B. pendula* (circle), and *C. betulus* (triangle) seedlings. ISO, isoprene; MBO, 2-methyl-3-buten-2-ol; MVK, methyl vinyl ketone; MAC, methacrolein; MEK, methyl ethyl ketone; MT, monoterpenes; SQT, sesquiterpenes. Color-codes indicate VOC related to the LOX (yellow), MEP (magenta), Shikimate (blue), and MVA (gray, i.e., SQT) pathways, and oxygenated VOCs (orange), respectively. See Supplementary Table 4 for PC 1–4 loadings, and Supplementary Figure 2 for species-specific PCAs.

**FIGURE 4:**
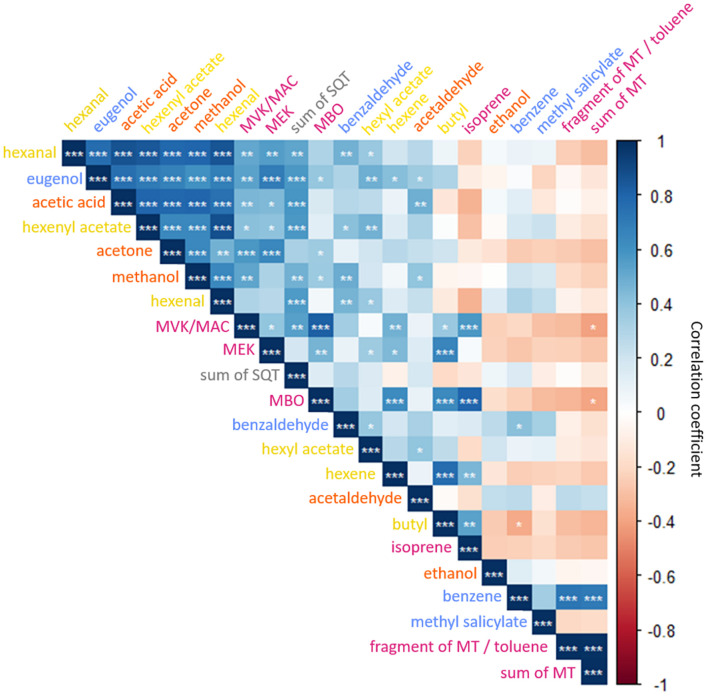
Matrix of Pearson correlation coefficients of 22 *m/z* assigned to constitutive VOCs of *Q. robur*, *F. sylvatica*, *B. pendula*, and *C. betulus* seedlings. Levels of significance among positive (blue) and negative (red) correlations are indicated as *p < 0.05, **p < 0.01, ***p < 0.001. ISO, isoprene; MBO, 2-methyl-3-buten-2-ol; MVK, methyl vinyl ketone; MAC, methacrolein; MEK, methyl ethyl ketone; MT, monoterpenes; SQT, sesquiterpenes. Color-code of VOC compounds indicates LOX (yellow), MEP (magenta), Shikimate (blue), and MVA (gray, i.e., SQT) pathways, and oxygenated VOCs (orange).

The detected emission spectra allow to speculate that a coordination between emission rates of specific VOCs, and thus potentially of stress responses, exist within and across pathways and catabolic reactions. To study the interrelation between emissions, a PCA was performed across species (Figure 3; PCAs per species can be found in Supplementary Figure 2). Pearson correlations were used to determine correlations between specific VOC emissions (Figure 4).

The two-dimensional space of the PCA ordinated the tree species according to the emissions: The emission composition spread from the most undifferentiated compound composition at the center to the most distinct in outer margins of the lowest compound variability. Spatially close vectors indicate tightly linked, potentially co-regulated VOC emissions. Principal component (PC) 1 separated emissions of hexyl acetate, eugenol, methanol, acetone, hexenal, and acetaldehyde from other VOCs, explaining 34.1% of the total variance. PC 2 separated emissions of monoterpenes, toluene (potentially summed with a MT fragment) and benzene from hexene, butyl, and isoprene, accounting for 19.9% of the total variance. PC 3 and 4 segregated monoterpene emissions and benzaldehyde from other VOCs, accounting for 10 and 8.6% of the total variance, respectively (see Supplementary Table 1 for details). A finer segregation by PC 1 and 2 was grouping hexanal, hexenyl acetate, acetic acid, and sesquiterpene emissions and MVK/MAC, MEK, and MBO emissions. Isoprene and its oxidation products MVK/MAC, MEK, and MBO, but also MTs and toluene (or fragment of MTs) were positively correlated (Figure 4). Methyl salicylate, ethanol, benzaldehyde, and hexyl acetate had small eigenvector values and therefore possess no strong links to other emitted VOCs (Supplementary Table 4).

Volatile organic compounds of the LOX pathway, i.e., hexenal, hexenyl acetate, and hexyl acetate, had a high covariance but did not correlate with hexene and butyl emission rates (Figure 4). Further, OVOCs were positively correlated among each other – except for ethanol. Emission rates of Shikimate pathway-related VOCs possessed no significant covariance among each other (Figure 4). VOCs related to either the LOX- and Shikimate pathways both correlated with the VOCs associated with MEP- and MVA pathways (Figure 4). In contrast, Shikimate-related benzene correlated with emitted monoterpenes, and butyl and hexene of the LOX-pathway with isoprene and OVOC emissions (Figures 3, 4). As the LOX- and Shikimate pathways are both upregulated during wounding and herbivore defense (Loreto and Schnitzler, 2010), it is likely that distinct correlations with other pathways could emerge under stress (Niinemets, 2010).

The segregation of isoprene, monoterpene, and sesquiterpene emissions were concomitant with the segregation of the emitting species, showing species-specific isoprene emissions for *Q. robur*, monoterpene emissions for *F. sylvatica* and sesquiterpene emissions for *B. pendula* (Figure 3). Although Vranová et al. (2013) outline that MEP- and MVA pathways share common metabolites and transcriptional factors, i.e., that MTs and SQTs can be synthesized from isopentenyl diphosphate (IPP) produced by both pathways, our results indicate that this shared precursor does not result in correlated emissions rates. This supports previous findings that isoprenoids synthesized *via* MEP- and MVA pathways are controlled by independent regulatory networks with restricted connectivity (Vranová et al., 2013). A closer look at VOCs synthesized by the MEP-pathway showed that high isoprene emissions are not associated with high monoterpene emissions (Figures 3, 4). This is supported by previous findings, showing that the MEP-pathway splits into either isoprene or monoterpene synthesis (Laule et al., 2003). Thus, a combined emission of isoprene and monoterpenes only occurs in species featuring isoprenoid-storing organs (Fineschi et al., 2013), which are neither found in *Q. robur* nor *F. sylvatica*. The synthesis of isoprene or monoterpenes suggests a species-dependent evolution with environmental stresses, e.g., as a response to high ozone levels and high temperatures (König et al., 1995; Oikawa and Lerdau, 2013).

Methanol emissions were highly correlated with sesquiterpenes, multiple VOCs from the LOX- and Shikimate pathways, and MVK/MAC and MBO (MEP pathway) emission rates (Figures 3, 4). No direct metabolic connection was previously reported for methanol and other VOCs (Loreto and Schnitzler, 2010; Oikawa and Lerdau, 2013; Dorokhov et al., 2018). However, emissions of methanol and GLVs of the LOX-pathway have both been shown to increase after damage or attacks by herbivores (Loreto and Schnitzler, 2010; Dorokhov et al., 2018). Further, we found acetone emissions closely linked with multiple VOCs including eugenol of the Shikimate pathway (Figures 3, 4); to the best of our knowledge, the latter has not been reported so far.

## Conclusion

Addressing tree seedlings grown under non-stress conditions, our work showed that the main constitutive VOC of *B. pendula* was methanol rather than sesquiterpenes. *Carpinus betulus* can be classified as a methanol and low monoterpene emitter rather than a close-to-zero emitter since its emission rates were similar to those of *F. sylvatica* and *B. pendula*. A PCA revealed a main segregation between isoprene, monoterpene and sesquiterpene/methanol emission, which each could be assigned to a tree species. Summarizing, the emission spectra of *C. betulus* was dominated by MeSA and, as for all other species, by VOCs of the MEP pathway and OVOCs. Clustered PCA-vectors and highly significant Pearson correlations of methanol, ethanol, acetic acid, and acetone illustrate strong co-emission patterns among OVOCs. The diverse volatile blends, with up to 18 co-emitted, quantifiable VOCs in *Q. robur*, have multiple potential functions, e.g., herbivory defense and antioxidants. As VOC emissions are highly depended on plant physiological status under local environmental conditions (Fitzky et al., 2019), more measurements of full emission profiles under stress conditions are key to advance our understanding of plant-environmental interactions under advancing climate change. In particular, potential changes of VOC emission blends in urban areas require attention due to high concentrations of air pollutants.

## Data Availability Statement

The raw data supporting the conclusions of this article will be made available by the authors upon request, without undue reservation.

## Author Contributions

AF, TK, MG, DT, HS, and BR: theory and conceptualization. AF, AP, LK, and MM: experimental measurements. AF, AP, LK, and MP: data analysis. AF: figures and tables. AF, HS, MP, and BR: writing of the manuscript. All authors jointly revised and approved the final version.

## Conflict of Interest

The authors declare that the research was conducted in the absence of any commercial or financial relationships that could be construed as a potential conflict of interest.

## Publisher’s Note

All claims expressed in this article are solely those of the authors and do not necessarily represent those of their affiliated organizations, or those of the publisher, the editors and the reviewers. Any product that may be evaluated in this article, or claim that may be made by its manufacturer, is not guaranteed or endorsed by the publisher.
