# Supplementary material for: Diversity and Interrelations Among the Constitutive VOC Emission Blends of Four Broad-Leaved Tree Species at Seedling Stage
Source: Front Plant Sci. 2021 Sep 24;12:708711. doi: 10.3389/fpls.2021.708711 (PMC8500217; doi:10.3389/fpls.2021.708711)
Supplement: Supplementary file 1 [file Data_Sheet_1.docx]

Diversity and interrelations among the constitutive VOC emission blends of four broad-leaved tree species at seedling stage

Anne Charlott Fitzky, Arianna Peron, Lisa Kaser, Thomas Karl, Martin Graus, Danny Tholen, Mario Pesendorfer, Maha Mahmoud, Hans Sandén, Boris Rewald

Supplementary Material and Methods

# Soil substrate

The used soil substrate was developed in Austria by the HBLFA (Höhere Bundeslehr- und Forschungsanstalt für Gartenbau) Schönbrunn in Vienna, the BAW (Bundesamt für Wasserwirtschaft) Petzenkirchen and MA 42 Wiener Stadtgärten, Vienna. The goal was to increase the water holding capacity, improve nutrient supply for city trees and increase the stability of the ground (for walkways and streets). The soil mixture was composed of 25% sand, 25% fine sediment form the Danube, 33.3% chippings and 16% compost. The mixture had an air capacity of > 15 Vol.%, a water holding capacity of >35 Vol.% and water permeability > 5 µm s^-1^ (Murer and Schmidt, 2015).

# Chamber design

| **A**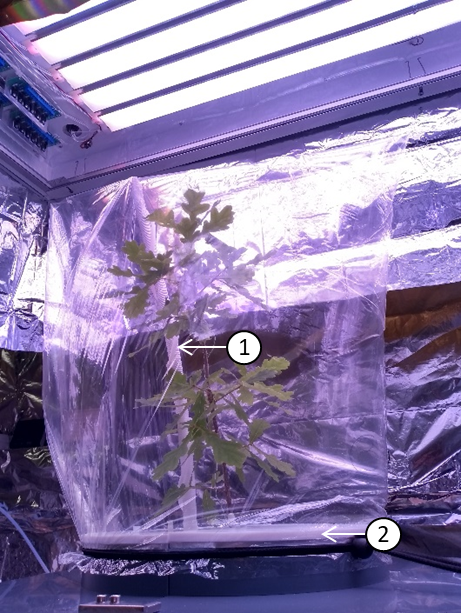 | **B**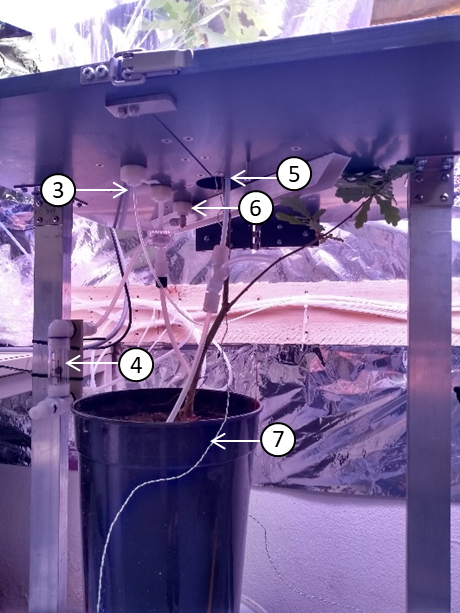 |
| --- | --- |

Supplementary Figure 1. VOC-chamber with enclosed tree. The upper part of the chamber (A) is showing the elevated inlet and PTFE plate (2), enclosing a tree in a PET bag with an LED light of 1450 µmol m^-^² s^-1^ light intensity at mid-canopy height. The lower part of the chamber (B) is showing the bottom half of the dividable table with the gas sampling line (3), the rotameter for down regulating the incoming air flow to 10 L min^-1^ (4), overflow (5), tubing for incoming air (6) through the elevated inlet (1), and the thermocouple wire (7).

# VOC emission calculations

The VOC concentrations from an enclosed tree in chamber 1 were corrected by subtracting the VOC concentrations of an empty chamber (chamber 2), this ensures that potential VOCs not scrubbed by the charcoal filter and/or released by the tubing, valves or chamber materials are considered as background concentrations.

$$\left[ VOC \right]tree=\left[ VOC \right]chamber 1 (with tree)-\left[ VOC \right]chamber 2 (no tree)$$

All measured VOC concentrations [VOC] were subsequently normalized and factored into VOC emissions (*E_0_*) per unit leaf area and flowrate (nmol m^-1^ s^-1^). Standardized VOC emissions (at 1000 µmol m^-^² s^-1^ and 30° C) were calculated following Guenther et al. (1993) using the light intensity at the middle of the canopy and the leaf temperature measured close to the VOC-sampling time point.

The standardized isoprene emission was calculated as:

$E_{ISO}={E_{0}}/{(C_{L}*C_{T})}$ (i)

where *E_ISO_* is the standardized isoprene emission (µg g^-1^_DW_ h^-1^), *E_0_* is the measured normalized isoprene emission (µg g^-1^_DW_ h^-1^), *C_L_* the light dependency factor and *C_T_* the temperature dependency factor (Guenther et al., 1993).

*M/z* 45 (acetaldehyde) is only light dependent (Jud et al., 2016) and was standardized using the light dependency factor *C_L_* as:

$E_{Ace}={E_{0}}/{C_{L}}$ (ii)

Standardized monoterpenes and sesquiterpene emissions (E_MT/SQT_) were calculated as:

$E_{MT/SQT}= {E_{0}}/{exp[\beta(T-T_{0})]}$ (iii)

where *E_MS_* is the emission rate (µg g^-1^_DW_ h^-1^) at *T_0_* (303.15 K), *E_0_* is either the measured monoterpene or the sesquiterpene emission (µg g^-1^_DW_ h^-1^) at the measured leaf temperature *T* (K) and empirical coefficient *β* at 0.1 (Guenther et al., 1991; Guenther et al., 1993).

Other measured VOCs were not standardized since they are not known to be light or temperature dependent.

# Determination of assimilation and transpiration rates, stomatal conductance and leaf area

Assimilation rate (*A,* µmol m^-^² s^-1^), transpiration rate (*E,* mol m^-^² s^-1^) and stomatal conductance (*g_s_,* mmol m^‑^² s^‑1^) during the VOC measurements were measured using a CIRAS-3 DC/SC infrared gas analyzer (PP Systems, Amesbury, MA, USA) with a sampling rate of 2 s, and calculated following the manufacturer’s instructions (PP-Systems, 2018).

$W=\left( \frac{F_{cuvette}}{60} \right)*\left( \frac{1}{V_{m}} \right)*(\frac{{10}^{4}}{leaf area})$ (iv)

where *W* is the flow (mol m^-^² s^-1^) entering the VOC-chamber, calculated with the flow rate of the chamber *F_cuvette_* (L min^-1^), the molar volume for ideal gas *V_m_* (L kPa K^-1^ mol^-1^) and leaf area (cm²) inside the VOC-chamber. The leaf area was measured the day after the VOC measurements. All leaves were removed and scanned with a flatbed scanner (Epson Expression 10000XL, Epson, Japan; 300 dpi) and green leaf area (cm^2^) was calculated using the program WinFOLIA 2013 Pro (Regent Instruments, Quebec, Canada).

The transpiration rate E (mol m^-^²s^-1^) was calculated accordingly (CIRAS-3 Operation Manual V. 2.01, PP-Systems):

$E=W* \frac{(H_{out}-H_{in})}{(p-H_{in})}$ (v)

where *H_out_* (mbar) is the partial pressure of water vapor inside the VOC-chamber and *H_in_* (mbar) the reference air supply entering the chamber. *p* is the ambient pressure (mbar). Assimilation rate *A* (µ mol m^-^² s^-1^) is calculated under consideration of *W* (mol m^-^² s^-1^) and *E* (mol m^-^²s^-1^) as follows:

$A= -\left[ {((C}_{out}- C_{in} \right)* W )+(C_{out}* E)]$ (vi)

The saturation vapor pressure *e_leaf_* (mbar) at leaf temperature *T_leaf_* (K) (Buck, 1981):

$e_{leaf}=6.1365*\exp\left( \frac{\left( T_{Leaf}*17.502 \right)}{(T_{Leaf}+240.97)} \right)$ (vii)

$r_{s}=\frac{e_{leaf}- H_{out}}{E*(p-\frac{e_{leaf}+ H_{out}}{2})}-r_{b}$ (viii)

$g_{s}=\frac{{10}^{3}}{r_{s}}$ (ix)

*E_leaf_* was used to calculate stomatal resistance *r_s_* (m² s mol^-1^) and stomatal conductance *g_s_* (mmol m^‑2^ s^‑1^) (Von Caemmerer and Farquhar, 1981) of the tree enclosed in the VOC-chamber. Given the set flow rates in relation to the chamber size, the boundary layer resistance to water vapor was approximated to be close to zero and subsequently neglected.

Supplementary Results

Supplementary Table 1. Stomatal conductance (g_s_, mmol m^-2^ s^-1^), net assimilation rate (A, µmol CO_2_ m^-2^ s^-1^), light intensity at mid-canopy height (µmol m^-2^ s^-1^) and leaf temperature (T_leaf_, °C) for *Q. robur, F. sylvatica, B. pendula* and *C. betulus* seedlings (mean ± SD). Stomatal conductance and A were calculated using measured CO_2_/H_2_O fluxes, see text for details. See Supplementary Table 2 for ANOVA on g_s_.

| **Species** | **g_s_** | | | **A** | | | **light intensity** | | | **T_leaf_** | | |
| --- | --- | --- | --- | --- | --- | --- | --- | --- | --- | --- | --- | --- |
|  | mmol m^-2^ s^-1^ | | | µmol m^-2^ s^-1^ | | | µmol m^-2^ s^-1^ | | | °C | | |
| *Quercus robur* | 94.18 | **±** | 30.97 | 6.82 | **±** | 1.22 | 1367.9 | **±** | 158.9 | 28.5 | **±** | 0.6 |
| *Fagus sylvatica* | 90.09 | **±** | 19.61 | 5.90 | **±** | 1.44 | 1400.3 | **±** | 129.3 | 28.1 | **±** | 0.4 |
| *Betula pendula* | 77.20 | **±** | 17.84 | 5.55 | **±** | 0.74 | 1325.7 | **±** | 132.2 | 28.7 | **±** | 1.4 |
| *Carpinus betulus* | 75.89 | **±** | 23.21 | 4.87 | **±** | 1.33 | 1306.6 | **±** | 126.0 | 28.6 | **±** | 1.1 |

**Supplementary Table 2.** ANOVA of stomatal conductance (g_s_), constitutive sesquiterpenes (SQT) and constitutive total VOC emission rates between seedlings of *Q. robur*, *F. sylvatica*, *B. pendula* and *C. betulus*. The data was log-transformed for normal distribution. Abbreviations: df – degree of freedom, SS – sum of square, MS – mean of square. See Supplementary Table 3 for group-wise comparisons of emission rates.

| **Source of variation** | **Factor** | **df** | **SS** | **MS** | ***F*-value** | ***p*-value** |
| --- | --- | --- | --- | --- | --- | --- |
| Species | g_s_ | 3 | 2018 | 672.8 | 1.223 | 0.32 |
| Species | SQT emissions | 3 | 10.83 | 3.611 | 3.036 | **0.0456** |
| Species | Total VOC emissions | 3 | 84.16 | 28.053 | 57.57 | **4.28e-12** |

**Supplementary Table 3.** Group-wise comparison (posthoc Tukey HSD test) of constitutive sesquiterpenes (SQT) and constitutive total VOC emission rates of *Q. robur*, *F. sylvatica*, *B. pendula* and *C. betulus* seedlings. The data was log-transformed for normal distribution. *n.s.* – not significant.

| **Species comparison** | **SQT emissions** | **total VOC emissions** |
| --- | --- | --- |
|  | ***p*-values** | |
| *C. betulus – B. pendula* | *n.s.* | <0.05 |
| *F. sylvatica - B. pendula* | *n.s.* | <0.05 |
| *Q. robur - B. pendula* | <0.05 | <0.001 |
| *F. sylvatica - C. betulus* | *n.s.* | *n.s.* |
| *Q. robur - C. betulus* | *n.s.* | <0.001 |
| *Q. robur - F. sylvatica* | *n.s.* | <0.001 |

**Supplementary Table 4.** Loadings of principal components (PC) 1 to 4 on the VOC emissions of seedlings of 4 tree species; see Figure 3 for corresponding PCA. VOC compounds are assigned to pathways or as oxygenated VOCs, see text for details. PC 3 isolated monoterpene emission from acetaldehyde from ethanol and explained 10% of the total variance. PC 4 isolated monoterpene and ethanol emissions and explained 8.6% of the total variance.

|  | **VOC compound** | **PC1** | **PC2** | **PC3** | **PC4** |
| --- | --- | --- | --- | --- | --- |
| **Oxygenated VOCs** | methanol | 0.62 | 0.05 | 0.01 | 0.01 |
|  | ethanol | 0.00 | 0.10 | 0.05 | 0.10 |
|  | acetaldehyde | 0.14 | 0.12 | 0.14 | 0.03 |
|  | acetic acid | 0.70 | 0.14 | 0.00 | 0.08 |
|  | acetone | 0.64 | 0.00 | 0.00 | 0.18 |
| **LOX pathway** | hexanal | 0.85 | 0.05 | 0.02 | 0.00 |
|  | hexenal | 0.58 | 0.20 | 0.02 | 0.01 |
|  | hexene | 0.17 | 0.34 | 0.09 | 0.04 |
|  | hexenyl acetate | 0.69 | 0.12 | 0.01 | 0.00 |
|  | hexyl acetate | 0.21 | 0.01 | 0.00 | 0.10 |
|  | butyl | 0.09 | 0.56 | 0.06 | 0.03 |
| **MEP pathway** | isoprene | 0.00 | 0.59 | 0.06 | 0.14 |
|  | MVK/MAC | 0.50 | 0.19 | 0.03 | 0.00 |
|  | MEK | 0.44 | 0.11 | 0.02 | 0.06 |
|  | MBO | 0.28 | 0.45 | 0.08 | 0.07 |
|  | sum of MT | 0.14 | 0.31 | 0.52 | 0.00 |
|  | fragment of MT/toluene | 0.09 | 0.31 | 0.58 | 0.00 |
| **MVA pathway** | sum of SQT | 0.35 | 0.08 | 0.00 | 0.01 |
| **Shikimate pathway** | benzene | 0.00 | 0.52 | 0.16 | 0.20 |
|  | benzaldehyde | 0.25 | 0.03 | 0.00 | 0.55 |
|  | methyl salicylate | 0.00 | 0.10 | 0.31 | 0.26 |
|  | eugenol | 0.77 | 0.00 | 0.05 | 0.02 |

| **A**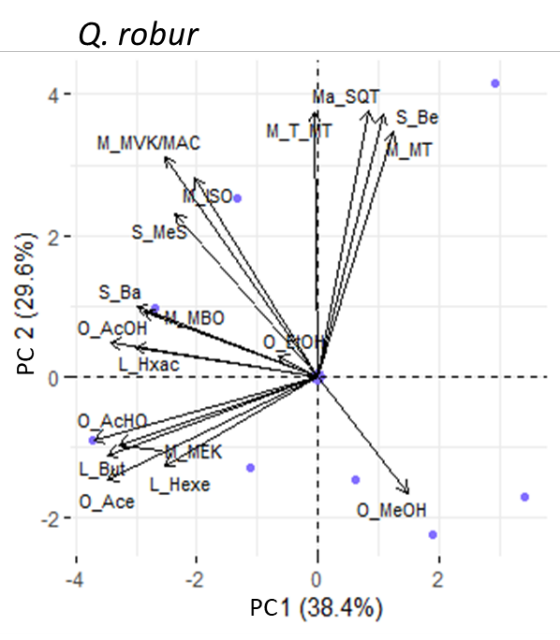 | **B**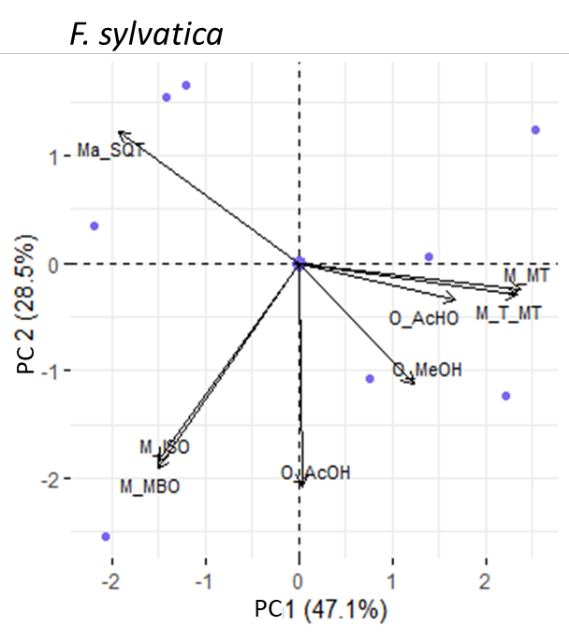 |
| --- | --- |
| **C**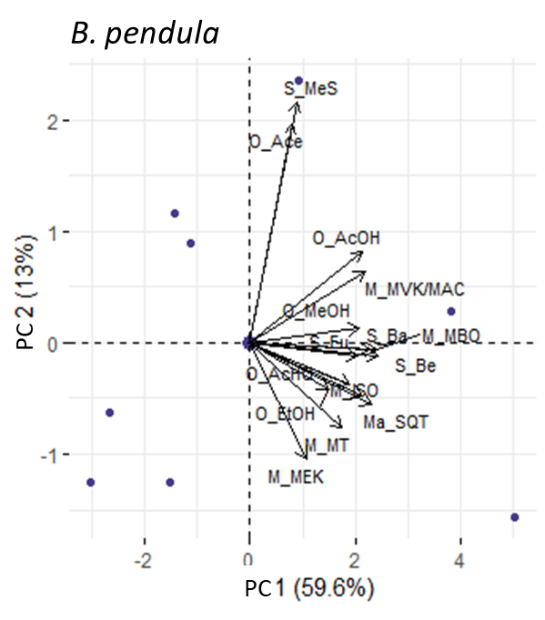 | **D**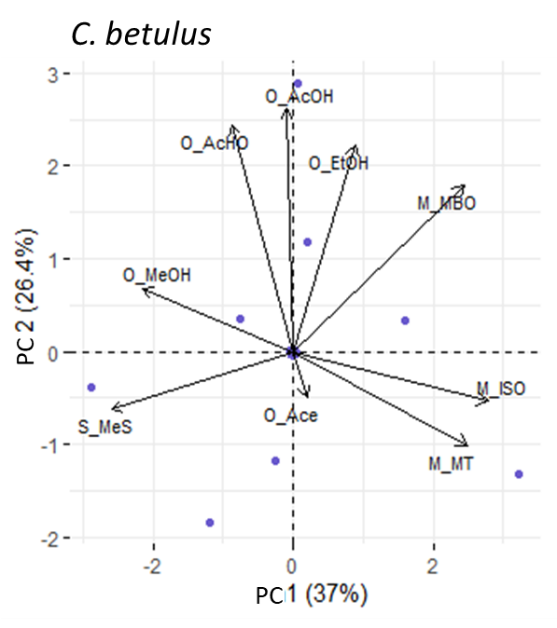 |

Supplementary Figure 2. Principle component analysis of VOC emissions of *Q. robur* (A), *F. sylvatica* (B), *B. pendula* (C), and *C. betulus* (D) seedlings, with PC 1 and 2 given. VOC of the LOX-pathway (L): L_But - butyl – *m/z*57.0335, L_Hexa - hexanal – *m/z*101.0961, L_Hexe - – hexenal – *m/z*99.0804, L_Hx - hexene – *m/z*85.1012, L_Hxa – hexenyl acetate – *m/z*143.1067, L_Hxac – hexyl acetate – *m/z*145.1223. VOC of the MVA-pathway (Ma): Ma_SQT - sum of sesquiterpenes – *m/z*205.1951. VOC of the MEP-pathway (M): M_F_MT - fragment of monoterpenes, toluene – *m/z*93.0699, M_ISO - isoprene - *m/z*69.0699, M_MBO - 2-Methyl-3-buten-2-ol - *m/z*87.0804, M_MEK - methyl ethyl ketone - *m/z*73.0648, M_MT - sum of monoterpenes – *m/z*137.1325, M_MVK/MAC - methyl vinyl ketone/methacrolein - *m/z*71.0491. Oxygenated VOCs (O): O_Ace - acetone – *m/z*59.0491, O_AcHO - acetaldehyde – *m/z*45.0335, O_AcOH - acetic acid – *m/z*61.0284, O_EtOH - ethanol – *m/z*47.0491, O_MeOH - methanol – *m/z*33.0335. VOCs of the Shikimate-pathway (S): S_Ba - benzaldehyde – m/z107.0491, S_Be - benzene – m/z79.0542, S_Eu - eugenol – m/z165.0916, S_MeS - methyl salicylate – m/z153.0546.

# Supplementary References

Buck, A.L. (1981). New equations for computing vapor pressure and enhancement factor. *Journal of Applied Meteorology and Climatology* 20(12)**,** 1527-1532. doi: 10.1175/1520-0450.

Guenther, A.B., Monson, R.K., and Fall, R. (1991). Isoprene and monoterpene emission rate variability: observations with eucalyptus and emission rate algorithm development. *Journal of Geophysical Research: Atmospheres* 96(D6)**,** 10799-10808. doi: 10.1029/91jd00960.

Guenther, A.B., Zimmerman, P.R., Harley, P.C., Monson, R.K., and Fall, R. (1993). Isoprene and monoterpene emission rate variability: model evaluations and sensitivity analyses. *Journal of Geophysical Research: Atmospheres* 98(D7)**,** 12609-12617. doi: 10.1029/93JD00527.

Jud, W., Vanzo, E., Li, Z., Ghirardo, A., Zimmer, I., Sharkey, T.D., et al. (2016). Effects of heat and drought stress on post‐illumination bursts of volatile organic compounds in isoprene‐emitting and non‐emitting poplar. *Plant, cell & environment* 39(6)**,** 1204-1215. doi: 10.1111/pce.12643.

Murer, E., and Schmidt, S. (2015). "Das Wiener Baumsubstrat - ein einfaches und tiefbaunahes Substrat für Stadtbäume". (Vienna: Österreichische Gesellschaft für Landschaftsarchitektur).

PP-Systems (2018). CIRAS-3 Operation Manual V. 2-01. Amesbury, MA, USA.

Von Caemmerer, S.v., and Farquhar, G.D. (1981). Some relationships between the biochemistry of photosynthesis and the gas exchange of leaves. *Planta* 153(4)**,** 376-387. doi: 10.1007/BF00384257.
